# Potential Rainwater Harvesting Improvement Using Advanced Remote Sensing Applications

**DOI:** 10.1155/2014/806959

**Published:** 2014-07-09

**Authors:** Mohamed Elhag, Jarbou A. Bahrawi

**Affiliations:** Department of Hydrology and Water Resources Management, Faculty of Meteorology, Environment & Arid Land Agriculture, King Abdulaziz University, Jeddah 21589, Saudi Arabia

## Abstract

The amount of water on earth is the same and only the distribution and the reallocation of water forms are altered in both time and space. To improve the rainwater harvesting a better understanding of the hydrological cycle is mandatory. Clouds are major component of the hydrological cycle; therefore, clouds distribution is the keystone of better rainwater harvesting. Remote sensing technology has shown robust capabilities in resolving challenges of water resource management in arid environments. Soil moisture content and cloud average distribution are essential remote sensing applications in extracting information of geophysical, geomorphological, and meteorological interest from satellite images. Current research study aimed to map the soil moisture content using recent Landsat 8 images and to map cloud average distribution of the corresponding area using 59 MERIS satellite imageries collected from January 2006 to October 2011. Cloud average distribution map shows specific location in the study area where it is always cloudy all the year and the site corresponding soil moisture content map came in agreement with cloud distribution. The overlay of the two previously mentioned maps over the geological map of the study area shows potential locations for better rainwater harvesting.

## 1. Introduction

Water cycle or the hydrological cycle assures that the quantity of water in the earth's environment under no circumstances changes, regardless of the state of the water as a liquid, gas, or solid state. Water repetitively circulates between the land, the oceans, and the atmosphere.

Adequate water management is founded on understanding the interconnections in the hydrological cycle. Informative knowledge of the designated catchment water balance is needed [1]. Catchment area by definition is the total area of terrestrial which catches rainfall and contributes the placid water to a certain surface water or potential groundwater recharge [2].

In semiarid regions climates, there is no accurate estimation of groundwater recharge. Existing estimation is based on the difference between the total amounts of rainfall and actual evapotranspiration due to indeterminate statistics of similar extents. Therefore no reliable information concerning absolute values of recharge can be obtained by the surface water balance [3, 4]. Recharge quantification problems from different sources are addressed by Gee and Hillel [5], Lerner et al. [6], Allison et al. [7], Stephens [8], Lerner [9], and Simmers [10], among others.

The impact of lithology and geomorphology in semiarid regions is exemplified by variances between designated areas and the corresponding geological feature [11–14]. Sinkholes in Saudi Arabia receive about 47% of the average rainfall (100 mm/year) and withdraw surface runoff into its sinkholes interconnections [15].

The formulation of cloud water is based on the interception of befalls droplets on different earth surface features including mainly the vegetation cover [16–20]. Lack of vegetation cover leads to insufficient cloud water formation and decrease in water precipitation into the soil in remarkable quantities [21]. Several elements stimulate the formation of cloud water interception. According to Elhag and Bahrawi [22], average cloud spatial distribution, droplet size, vegetation cover, and wind velocity are basically encountered. Presence of mountainous chain and precipitous slopes in a designated area are the origination of what is so called the cloud belt; cloud interception in the study area is expected to be a joint phenomenon along the area [23].

Clouds exert a dominant influence on solar energy absorbed by the earth and on infrared radiation emitted to space. It is known that clouds present a problem; they act to cool the planet by reflecting solar radiation to space and warm the planet by reducing radiation emitted to space [24–26]. Accurate detection of clouds from remote sensing images are with a major concern for a wide range of remote sensing applications, especially by sensors detecting ultraviolet (UV) and visible and near-infrared (VNIR) range of the electromagnetic spectrum [27, 28].

To optimize the use of limited water resources in arid environments unconventional methods of planning are required [23]. Soil moisture monitoring is a crucial feature of managing water requirements of agricultural fields founded on advanced irrigation techniques [29].

The aim of the current study is to examine the interconnection between spatiotemporal distribution of the conducted cloud likelihood maps and clouds underneath terrain features to improve potential rainwater harvesting in the study area.

## 2. Materials and Methods

### 2.1. Study Area

**Figure 1:**
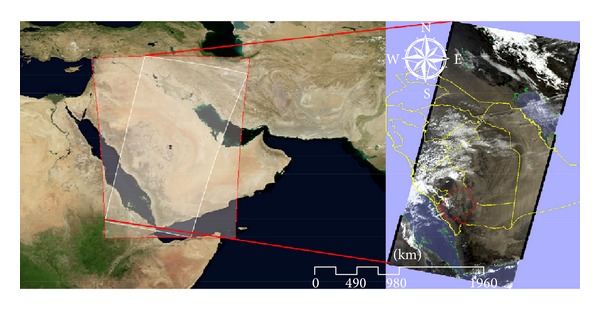
Administrative boundaries of KSA regions with location of the study area highlighted.

Asir region is located at the southwest of Saudi Arabia (Figure 1). Asir consists of about 100,000 km^2^ of Red Sea coastal plains and high mountains, and the upper valleys of the wadis (seasonal watercourses) are Bīshah and Tathlīth. Asir is a prosperous agricultural region. It has an area of 77,088 km² and an estimated population of 1,563,000. It shares a short border with Yemen. Its capital is Abha. The average annual rainfall in the highlands probably ranges from 300 to 500 mm falling in two rainy seasons, the chief one being in March and April with some rain in the summer. Temperatures are extreme, with diurnal temperature ranges in the highlands being the greatest in the world. It is common for afternoon temperatures to be over 30°C, yet mornings can be frosty and fog can cut visibility to near zero percent. As a result, there is much more natural vegetation in Asir than in any other part of Saudi Arabia.

**Figure 2:**
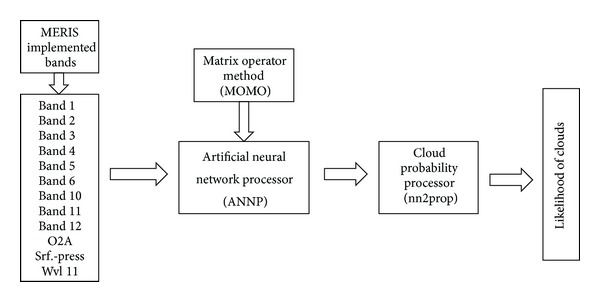
Cloud detection algorithm.

General structure of the cloud detection algorithm is illustrated in Figure 2. During development of the algorithm by Fischer and Grassl [30] and Fell and Fischer [31], using the radiative transfer model MOMO (matrix operator method), simulated cloud and noncloud top of atmosphere radiance have been produced and an artificial neural net has been trained. Thus, artificial neural network is now used in the cloud probability processor, where it is fed with the reflectances and the pressure as shown in Figure 2. Postprocessing is applied after the net (nn2prop) which scales the output of the artificial neural network into a probability value.

### 2.2. Methodological Framework

#### 2.2.1. Algorithm Basics

According to Lindstrot et al. [32], clouds are easy to detect when a manual classification of satellite images is done; their automatic detection is difficult. Clouds have four special radiative properties that enable their detection: (1) clouds are white, (2) clouds are bright, (3) clouds are higher than the surface, and (4) clouds are cold. However clouds, as the most variable atmospheric constituent, seldom show all four properties at the same time.

Thin clouds show a portion of the underlying surface spectral properties, and low clouds are sometimes warm. Also, some surface types like snow and ice have spectral properties that are similar to some of the cloud properties. Therefore simple thresholding algorithms often fail, and existing cloud detection schemes use several different cascaded threshold based tests to account for the complexity [33–35].

#### 2.2.2. Algorithm Specification

The cloud probability algorithm has been developed and implemented by Free University Berlin and Brockmann Consult. It is also used in the Global MERIS Land Albedo Map project [36]. The cloud probability algorithm is using nine spectral bands of MERIS. Specifically, the ratio of band 10 (cloud optical thickness and cloud-top pressure reference), band 11 (Cloud-top/Surface pressure) and band 12 (aerosol, vegetation); which is an oxygen absorption indicator. According to the European Centre for Medium-Range Weather Forecasts (ECMWF), surface pressure and the exact wavelength of band 11 used as algorithm input parameters. As an output, it yields a probability value (0 to 1) indicating if a pixel can be regarded as a cloud or not. Such a probability permits a more flexible way to work with identified clouds compared to a binary cloud mask. The algorithm uses two different artificial neural networks.

MERIS measures radiances in 15 channels between 400 nm and 1000 nm. Thus the very valuable thermal information and information about the liquid and ice water absorption at 1.6 *μ*m and 3 *μ*m are not available. The cloud detection for MERIS therefore relies on bands 10, 11, and 12 according to Lindstrot et al. [32]. In addition a slight absorption of snow at 900 nm could be used to discriminate snow from low clouds [36].

Watershed delineations and its companions of DEM analyses are processed under GIS environment using conventional methods.

#### 2.2.3. Cell Statistics

**Figure 3:**
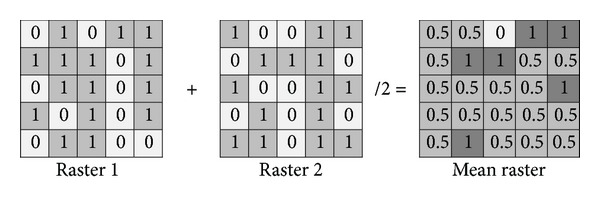
Mean command illustration.

Under GIS environments [37], cell statistics calculates a per-cell statistic from multiple rasters (59 rasters), in the current case the “Mean” command which calculates the average of all input raster values as illustrated in Figure 3. Resulting cloud average distribution is then converted into percentages raster based on 0 and 1 cloud probability. Classifying the final spatiotemporal cloud average distribution map was based on Jenks rule of classification, where the output classes were based on natural groupings innate in the data [37]. Jenks rule identifies break points by picking the class breaks that best group similar values and heighten the differences between classes. The features were divided into classes whose boundaries were set where there were fairly big jumps in the data values. The final output map was divided into three classes:

- not cloudy,
- marginally cloudy,
- cloudy.

#### 2.2.4. NDWI Calculation

The Normalized Difference Water Index (NDWI) is a satellite index derived from near-infrared (NIR) and short-wave infrared (SWIR) channels:

(1) $$\begin{array}{c} \text{NDW}\;\text{I}=\frac{\left(1-\text{SWIR}/\text{NIR}\right)}{\left(1+\text{SWIR}/\text{NIR}\right)}. \end{array}$$

The amount of water present in leaf internal structure mainly affects the spectral reflectance in the SWIR interval (1.2–1.7 *μ*m). The SWIR reflectance is also sensitive to leaf internal structure. Because the NIR is affected by leaf internal structure and leaf dry matter, but not by the water content, the combination of both into NDWI “removes” leaf dry matter and internal structure. NDWI is less susceptible to atmospheric scattering than NDVI but does not remove completely the background soil reflectance effects, similar to NDVI. Because the information about vegetation canopies contained in the SWIR channel is very different from that contained in the VIS channel, NDWI should be considered as an independent vegetation index.

## 3. Results and Discussion

**Figure 4:**
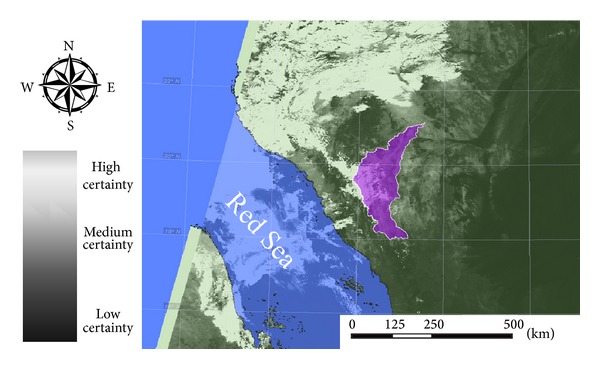
Cloud certainty map with watershed highlighted.

Cloud average distribution map over the designated area was performed under the tropical atmosphere case of artificial neural network implementation [32, 38]. Cloud probability maps are configured within three levels of certainty as illustrated in Figure 4. Levels of certainty are (A) more than 80% of cloud probability (cloudy), (B) from 80 to 20% cloud probability (marginally cloudy), and (C) less than 20% cloud probability (not cloudy). The algorithm implementation conducted robust results over the study area with high accuracy cloud maps under correct sky conditions; the algorithm maintains successfully high precision by 75% [39].

**Figure 5:**
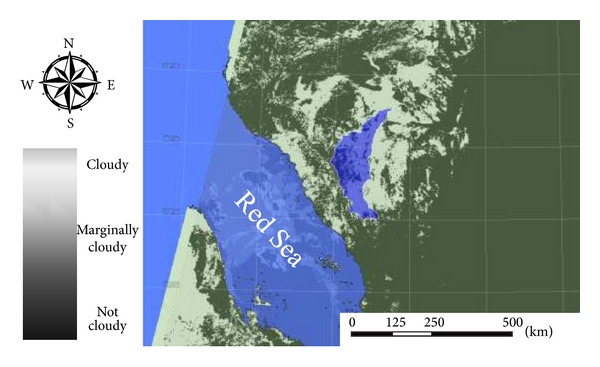
Cloud probability classes with watershed highlighted.

**Figure 6:**
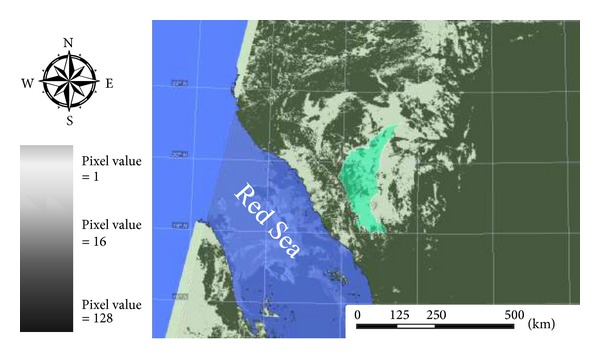
Cloud probability classification flags used with watershed highlighted.

Certainty levels were converted into three cloud probability classes as shown in Figure 5. According to Table 1, most of the used flags belong to suspect pixels (value of 8) and to overland pixels (value of 16; Figure 6) to confirm the reliability of the algorithm performance over the designated study area which is mainly an agricultural land and desert [40, 41]. In Figure 6, proper selection of threshold value according to Table 1 resolved into significant differences between cloud free water and cloudy water pixels. Therefore, the clear pixels could be separated from cloudy pixels. However, this also indicates that the discrimination between land and sea by using the image of brightness temperature is successful [42, 43].

Resulting cloud maps of the 59 MERIS images are then classified into two classes only to perform the average command: (1) certain cloudy pixel (<80% accuracy) and (2) not cloudy pixels (>80% accuracy); values of one and zero are assigned to the cloud classes, respectively.

**Figure 7:**
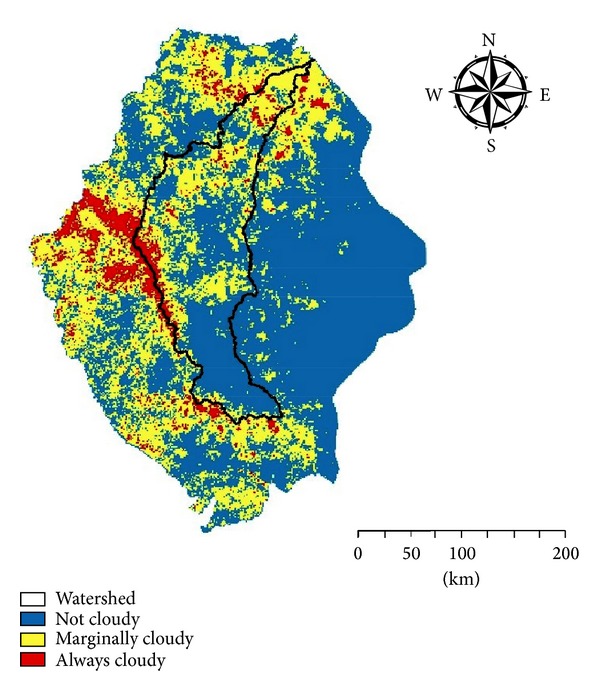
Cloud average distribution map over the southern part of KSA.

The current algorithm proved to be efficient in cloud detection over agricultural land and desert [40, 41, 44, 45]. Cloud average distribution map over Asir region, southern KSA, illustrated in Figure 7 confirms that the majority of the study area is described generally to be a cloud-free area most of the year. Formulation of trapped clouds due to the mountain belt located in the study area maintains cloudy cover area most of the year. Watershed delineation resulted into several watershed exists in the study area; only the biggest one is represented in Figure 7. The western side of the watershed shares cloudy coverage most of the year with the mountain belt. This cloudy cover might be considered as source of the watershed torrents [46]. Cloud spatiotemporal distribution map pointed out that the majority of the watershed lies under either marginally cloudy or not cloudy areas. However, the sink of the watershed is covered mostly by clouds.

**Figure 8:**
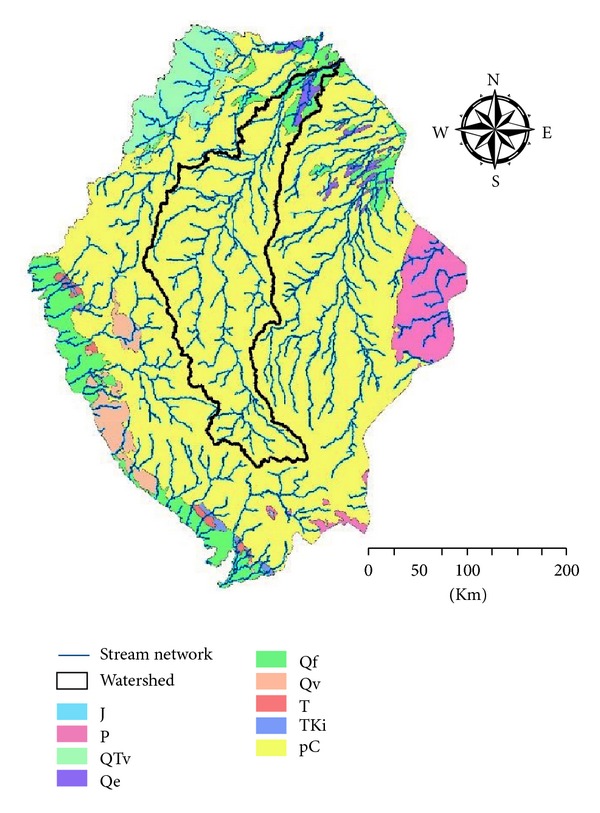
Stream network confined in the main watershed placed over the geology map of the southern part of KSA.

Selected watershed located within Precambrian geological feature which is not adequate for groundwater recharge purposes due to the permeability of Precambrian layer; the watershed is deliberated as low permeable layer [12–14]. On the contrary, only a small lower part of the watershed lies over a Quaternary alluvial geological layer which is characterized by higher permeability [12–14]; the sink of the watershed receives the runoff and settles it down leaving a better chance for groundwater recharge process (Figure 8).

**Figure 9:**
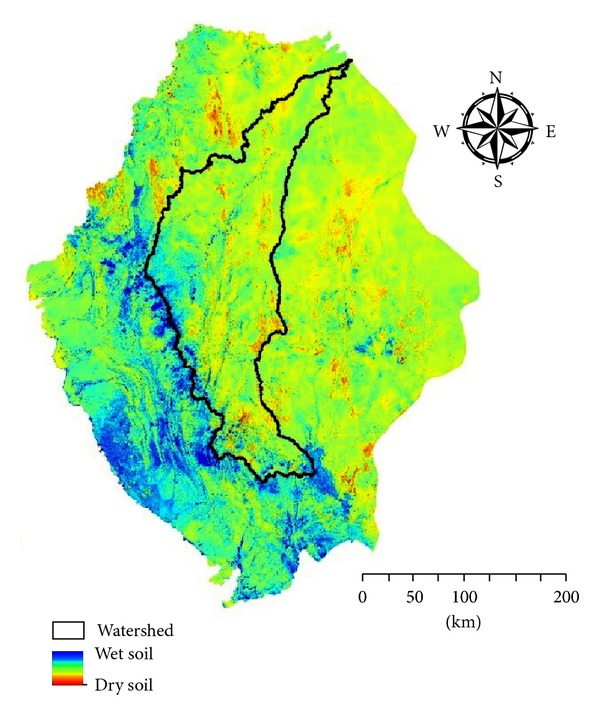
Remote sensing based soil moisture content map of the southern part of KSA.

Valuable information could be extracted from remote sensing data only when the limitation conditions are taken into account. Limiting conditions for the application of Normalized Difference Water Index may rely mainly on surface roughness [47] and the type of land use [48]. Normalized Difference Water Index exemplified in Figure 9 indicates that most of eastern mountain belt of the study area including the designated watershed is located over a relatively dry soil. Dry soils have a higher tendency to accommodate preferably surface water which may lead to improved groundwater recharge [14, 49–51]. Differences in spatial soil moisture content maps can be used for the identification of distinctive areas of potential for groundwater recharge [3].

**Figure 10:**
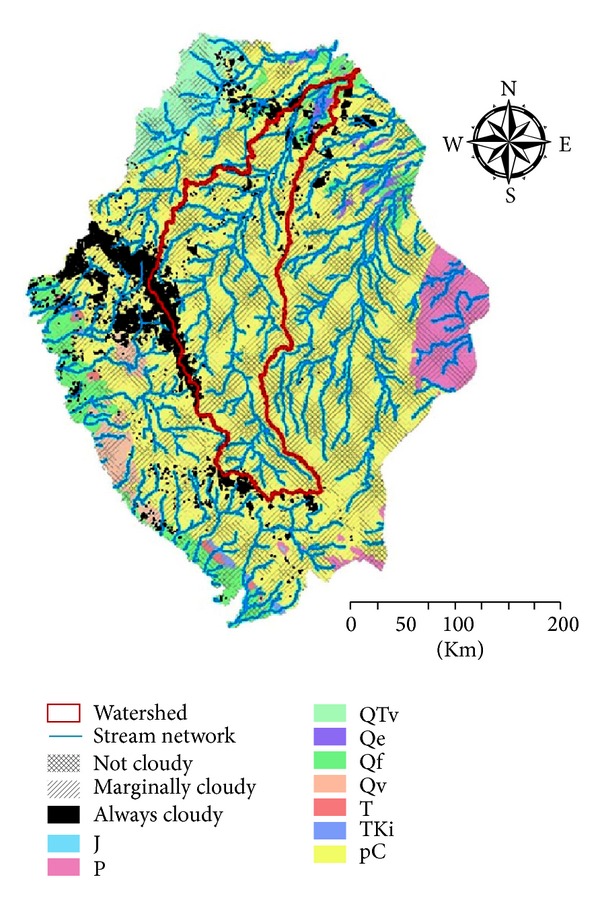
Cloudy areas within the watershed placed over the geology map of the southern part of KSA to locate areas suitable for potential groundwater recharge.

The finding of the current research is based on the interconnections between the previously conducted results as composed in Figure 10; cloud average distribution map intersected with the geological map of the designated area. Furthermore the stream network within the main watershed of the area draws attention to the watershed sink. The sink is characterized by cloudy sky most of the year, relatively semidry soil, and adequate geological permeable layer. The interconnections of those conditions improve groundwater recharge process through less evaporation effect, slower saturation velocity, and higher potential permeability, respectively [52–54].

## 4. Conclusions and Recommendations

Rainwater harvesting and conservation are the activity of direct collection of rainwater. The conservation of rainwater so collected can be stored for direct use or can be recharged into groundwater. The aim of the present work is to apply the cloud probability algorithm developed by the Institute for Space Science, Free University Berlin. Performing the algorithm resulted in robust cloud probability maps over the designated area. Classifying the resulting maps into two classes, cloudy and not cloudy, eases the sum of all the cloudy pixels of the 59 probability maps conducted. The spatiotemporal distribution of the clouds raises the quest for the proper use of such a method. The correlation between the cloudy pixels and land use land cover beneath is the keystone of proper practice of the current approach. As the clouds are the main source of precipitation, using the cloud probability maps will be strongly correlated to water resources management in the area. The practices of water resources management are many but the present methodology helps decision makers to decide where the dams need to be built to increase the potentials of groundwater recharge as a direct implementation of the adopted method. However, several applications of integrated water resources management or risk assessments may benefit from the current method, that is, estimation of soil moisture content, improvement of rainfed agriculture, and/or production of risk maps to avoid the drastic results of flooding events that may occur. Further work on the correlation between the cloud probability maps and land use land cover beneath may need to be carried out.

## Figures and Tables

**Table 1:** Cloud probability maps corroborated flags of MERIS imagery.

Name	Value	Description
Cosmetic	1	Pixel is cosmetic
Duplicated	2	Pixel has been duplicated (filled in)
Glint_Risk	4	Pixel has glint risk
Suspect	8	Pixel is suspect
LAND and/or OCEAN	16	Pixel is overland, not ocean
Bright	32	Pixel is bright
Coastline	64	Pixel is part of a coastline
Invalid	128	Pixel is invalid
